# Artificial Intelligence Algorithms to Assess Hormonal Status From Tissue Microarrays in Patients With Breast Cancer

**DOI:** 10.1001/jamanetworkopen.2019.7700

**Published:** 2019-07-26

**Authors:** Gil Shamai, Yoav Binenbaum, Ron Slossberg, Irit Duek, Ziv Gil, Ron Kimmel

**Affiliations:** 1 Department of Electrical Engineering, Technion Israel Institute of Technology, Haifa, Israel; 2 Laboratory of Pediatric Oncology, Tel Aviv Sourasky Medical Center, Tel Aviv, Israel; 3 Laboratory for Applied Cancer Research, Rambam Healthcare Campus, Rappaport Institute of Medicine and Research, Haifa, Israel; 4 Departmemt of Computer Science, Technion Israel Institute of Technology, Haifa, Israel; 5 Department of Otolaryngology–Head and Neck Surgery, Rambam Health Care Campus, Haifa, Israel

## Abstract

**Importance:**

Immunohistochemistry (IHC) is the most widely used assay for identification of molecular biomarkers. However, IHC is time consuming and costly, depends on tissue-handling protocols, and relies on pathologists’ subjective interpretation. Image analysis by machine learning is gaining ground for various applications in pathology but has not been proposed to replace chemical-based assays for molecular detection.

**Objective:**

To assess the prediction feasibility of molecular expression of biomarkers in cancer tissues, relying only on tissue architecture as seen in digitized hematoxylin-eosin (H&E)–stained specimens.

**Design, Setting, and Participants:**

This single-institution retrospective diagnostic study assessed the breast cancer tissue microarrays library of patients from Vancouver General Hospital, British Columbia, Canada. The study and analysis were conducted from July 1, 2015, through July 1, 2018. A machine learning method, termed *morphological-based molecular profiling* (MBMP), was developed. Logistic regression was used to explore correlations between histomorphology and biomarker expression, and a deep convolutional neural network was used to predict the biomarker expression in examined tissues.

**Main Outcomes and Measures:**

Positive predictive value (PPV), negative predictive value (NPV), and area under the receiver operating characteristics curve measures of MBMP for assessment of molecular biomarkers.

**Results:**

The database consisted of 20 600 digitized, publicly available H&E-stained sections of 5356 patients with breast cancer from 2 cohorts. The median age at diagnosis was 61 years for cohort 1 (412 patients) and 62 years for cohort 2 (4944 patients), and the median follow-up was 12.0 years and 12.4 years, respectively. Tissue histomorphology was significantly correlated with the molecular expression of all 19 biomarkers assayed, including estrogen receptor (ER), progesterone receptor (PR), and *ERBB2* (formerly *HER2*). Expression of ER was predicted for 105 of 207 validation patients in cohort 1 (50.7%) and 1059 of 2046 validation patients in cohort 2 (51.8%), with PPVs of 97% and 98%, respectively, NPVs of 68% and 76%, respectively, and accuracy of 91% and 92%, respectively, which were noninferior to traditional IHC (PPV, 91%-98%; NPV, 51%-78%; and accuracy, 81%-90%). Diagnostic accuracy improved given more data. Morphological analysis of patients with ER-negative/PR-positive status by IHC revealed resemblance to patients with ER-positive status (Bhattacharyya distance, 0.03) and not those with ER-negative/PR-negative status (Bhattacharyya distance, 0.25). This suggests a false-negative IHC finding and warrants antihormonal therapy for these patients.

**Conclusions and Relevance:**

For at least half of the patients in this study, MBMP appeared to predict biomarker expression with noninferiority to IHC. Results suggest that prediction accuracy is likely to improve as data used for training expand. Morphological-based molecular profiling could be used as a general approach for mass-scale molecular profiling based on digitized H&E-stained images, allowing quick, accurate, and inexpensive methods for simultaneous profiling of multiple biomarkers in cancer tissues.

## Key Points

**Question:**

Can molecular markers of cancer be extracted from tissue morphology as seen in hematoxylin-eosin–stained images?

**Findings:**

In this diagnostic study of tissue microarray hematoxylin-eosin–stained images from 5356 patients with breast cancer, molecular biomarker expression was found to be significantly associated with tissue histomorphology. A deep learning model was able to predict estrogen receptor expression solely from hematoxylin-eosin–stained images with noninferior accuracy to standard immunohistochemistry.

**Meaning:**

These results suggest that deep learning models may assist pathologists in molecular profiling of cancer with practically no added cost and time.

## Teaser

This single-institution diagnostic study uses a machine learning method (morphological-based molecular profiling) to assess the prediction feasibility of molecular expression of biomarkers in cancer tissue of patients with breast cancer.

## Introduction

Since the birth of modern pathology, identification of molecular markers in tissues has relied on chemical processes. Immunohistochemistry (IHC) using monoclonal antibodies has become the workhorse of molecular phenotyping, despite its marked limitations: it is time consuming, costly, and highly dependent on tissue handling protocols, reagents, and expert laboratory technicians. Moreover, interpretation of the results is primarily visual and relies on pathologists’ subjective interpretation.^1,2,3,4^

Artificial intelligence and machine learning technology are gaining ground in medicine because of their unmatched ability to make accurate predictions. In pathology, machines that quickly identify distinctive histomorphological features can now differentiate between neoplastic and nonneoplastic lesions,^5,6,7^ identify metastasis in lymph nodes,^8^ and perform tumor grading.^9^ Machines have been shown to predict clinical data from biopsy images by identifying morphological features that were unseen by humans.^5,10^ As such, Beck et al^11^ showed that the prognosis of patients with breast cancer, traditionally determined by a clinicopathologic multifactorial model, could be predicted from hematoxylin-eosin (H&E)–stained histological images of cancer specimens by using machine learning.

We explored whether the molecular profile of cancer is encoded in histomorphological structures that are beyond human apprehension. For this task, we applied machine learning methods to a process we term *morphological-based molecular profiling* (MBMP) for robust determination of molecular expression based on H&E-stained images. We then applied MBMP on a publicly available archive of breast cancer specimens to explore the associations between features in tissue morphology and expression of multiple molecular biomarkers.

With the advantages of a digital method, MBMP may be able to address innate problems of traditional molecular profiling techniques. In breast cancer, for example, an estimated discrepancy as high as 19% is reported for estrogen receptor (ER) estimation by central or peripheral laboratories, when using different antibody clones, or when following various tissue-processing protocols.^12,13,14,15,16^ Automated digital methods could eliminate some of these problems and improve diagnostic accuracy and patient care. Once established, MBMP could be trained to simultaneously predict the expression of multiple biomarkers, thus allowing a global approach for mass-scale biomarker expression prediction. By portraying molecular pathways that drive cancer progression from a completely different perspective, MBMP might provide an additional tool for personalized treatment tailoring against cancer.

## Methods

### Ethical Review and Reporting Guideline

This study was based on data made publicly available by the Genetic Pathology Evaluation Centre, Vancouver, British Columbia, Canada. All research at the Genetic Pathology Evaluation Centre is performed in accordance with institutional and provincial ethical guidelines. Because the data did not include patient contact or medical record review, informed consent was not required. This study follows the Standards for Reporting of Diagnostic Accuracy (STARD) reporting guideline.

### Data Processing and Participants

The database was composed from a publicly available tissue microarray (TMA) library, published by Genetic Pathology Evaluation Centre. All data can be found on http://bliss.gpec.ubc.ca/ (libraries 01-011 and 02-008), http://www.gpecimage.ubc.ca/, and https://tma.im/tma_portal/C-Path/. Details about the scanner, image resolution, eligible patients, and cut points used in this work can be found in eMethods 1 and eTable 1 in the Supplement.

### Exploring Correlations: Experimental Design Overview

To explore whether correlation exists between the morphological features of the tumor and molecular biomarker expression, we developed a learning-based model for automatic analysis of TMA images (eFigure 1 in the Supplement). In this model, the image was first divided into small regions termed *superpixels*.^17^ Second, within each superpixel, different local arithmetic operations were performed using a feature extraction pipeline (eFigure 2 in the Supplement). Next, we calculated a global mean across each local feature to obtain a set of features per image. Because each patient had multiple TMA images, the mean of these features was calculated across the images to obtain a set of 1296 features per patient. Finally, an L_1_ regularized logistic regression was trained to predict the dichotomized molecular biomarker expression (positive or negative) of a molecule in question from the feature vector. When training the classifier, we balanced the data by replicating the minority class of patients.

### Predicting Molecular Expression: Experimental Design Overview

We adapted a state-of-the-art deep convolutional neural network (CNN) to predict dichotomized molecular expression solely from H&E-stained histological images. The proposed model was based on the residual network (ResNet)^18^ architecture (eFigure 3A in the Supplement) and was trained to predict the molecular expression from a single H&E-stained image. The ResNet unit takes a 512 × 512-pixel H&E-stained image as an input without any preprocessing and produces 64 features that encode it. Unlike the feature extraction pipeline, these features are not constrained to predefined arithmetic operations. Alternatively, the ResNet learns the operations that are optimized to the set goal. We used 2 ResNet units to construct an inference pipeline (eFigure 3B in the Supplement). Given an H&E-stained image, 64 features were produced from each ResNet and concatenated into a set of 128 features. These features replaced the feature extraction pipeline presented in eFigure 2 in the Supplement. As before, an L_1_ regularized logistic regression was trained to predict the molecular expression from the features.

The inference pipeline outputs a score *r* per image that represents the probability that the molecule in question is expressed. For patients with multiple TMA images, we calculated the mean of the features across all images to obtain a per-patient *r* score (details in eMethods 2 in the Supplement). We defined *T_l_* and *T_h_* as low and high thresholds, respectively, holding the condition 0 < *T_l_* ≤ *T_h_* < 1, that can be tuned to adjust the confidence of the prediction. The molecular expression was predicted as negative for *r* *<* *T_l_* and positive for *T_h_* *<* *r*, whereas cases with *T_l_* < *r* < *T_h_* were considered inconclusive. A larger gap between the thresholds is likely to improve the specificity and sensitivity of the system at the expense of increasing the percentage of inconclusive classifications. We experimented with different settings of thresholds and show the results in the Results section.

### Implication of the Data Set on the System’s Performance

We characterized the association between the prediction performance and the database used in terms of cohort size, image resolution, number of TMA images per patient, and image cut size. To this end, we randomly selected a subset of patients from cohort 2. We changed the resolution and cut size of the H&E-stained images and the number of TMA images per patient used for analysis (details in eMethods 3 in the Supplement). We used the feature extraction pipeline to extract features and predict the expression of Ki-67, ER, PR, and *ERBB2* (formerly *HER2*). We then repeated only the TMA-images-per-patient experiment using the CNN-based pipeline for ER status prediction for both cohorts.

### Response Maps

One of the major limitations of CNNs is that the learning procedure can be considered a “black box” in the sense that tracking down the intuition behind it might be impossible. To shed light on the learning mechanism, we designed our CNN to produce a response map that revealed the contribution of each area in the H&E-stained image to the final predicted *r* score (eFigure 4 in the Supplement).

### The MBMP Process

Morphological-based molecular profiling is a CNN-based image analysis protocol that is aimed to predict molecular expression from H&E-stained specimens. The process described in the Methods section consists of the following 4 stages: data collection, training of the primary network, training of the validation network, and a final inference and decision stage (full description in eMethods 4 in the Supplement).

### Statistical Analysis

Data were collected and analyzed from July 1, 2015, through July 1, 2018. We used the area under the receiver operating characteristics curve (AUC), accuracy, balanced accuracy, positive and negative predictive values, and *P* < .01 with a 1-tailed hypothesis test indicating statistical significance as our statistical measures. The receiver operating characteristics curves were plotted as sensitivity vs specificity. Balanced accuracy is defined as the mean of sensitivity and specificity and is a useful measure when data are imbalanced. Likelihood ratio χ^2^ tests and *P* values for multiple logistic regression and associations for stratification by percentage of ER-positive cells were performed using the likelihood-ratio test in JMP software, version 14.0 (SAS Institute Inc). The Bhattacharyya distance^19^ (D_BC_) was used to measure similarity between distributions. The logistic regression was implemented using the Glmnet package in Matlab, version R2013B (MathWorks).

## Results

### Participants and Database

The database originated from 2 cohorts, including a total of 5356 patients with breast cancer who had 20 600 digitized H&E-stained histological images. Cohort 1 (library 01-011) included 412 patients. Each patient had 14 H&E-stained TMA images and annotations for ER expression. Some of the images have masks segmenting epithelial and stromal compartments.^11,20^ Cohort 2 (library 02-008) included 4944 patients. Each patient had 3 H&E-stained TMA images, 1 IHC-stained TMA image for ER using SP1 antibody, and annotations for 19 biomarkers. The median age at diagnosis was 61 years for cohort 1 and 62 years for cohort 2, and the median follow-up was 12.0 years and 12.4 years, respectively.

### Association Between Biomarker Expression and Tumor Morphology

We used the proposed model to extract features from each patient in cohort 2. We assessed the correlations between tumor morphology, encoded as the extracted features, and the expression of 19 distinct biomarkers by 10-fold cross-validation, in terms of accuracy, balanced accuracy, and *P* value. For all 19 biomarkers evaluated, the output prediction scores were significantly correlated with the molecular expression (eTable 2 in the Supplement). The prediction performance did not broadly differ for markers expressed at the nucleus (Ki-67 and ER), the cytoplasm, or the plasma membrane (epidermal growth factor receptor and proto-oncogene tyrosine-protein kinase receptor Ret). In addition, markers expressed at the tumor stroma (FOXP3 and CD8) or epithelial compartments (PR and insulinlike growth factor type 1 receptor) had no noticeable difference. Understandably, Ki-67 scored highest, because its expression is associated with high-grade tissue architecture that is easily distinguishable by pathologists and machines.^21,22^ Unexpectedly, FOXP3 and CD8, immune markers less obviously associated with distinctive morphology, also received high prediction accuracies. This analysis showed that the expression of molecular markers is phenotypically reflected as subtle motifs in tissue morphology. These previously unobserved patterns were identified by a suited learning model, suggesting that artificial intelligence could be used to predict molecular expression directly from H&E-stained images.

### Predicting ER Expression

To investigate the possibility of biomarker expression prediction from tissue histomorphology, we trained the proposed CNN model to predict the expression of ER from H&E-stained histological images. We chose to experiment on ER owing to its significance in breast cancer and its large representation in the available data, that is, 19 331 H&E-stained images of 4933 patients in both cohorts (eTable 1 in the Supplement). Recent studies with robust anti-ER antibodies suggested that the subgroup of ER-negative/PR-positive tumors does not actually exist and represents false-negative IHC stain interpretations.^23^ To improve the credibility of the evaluation, this equivocal subgroup of patients was omitted from the primary analysis (85 of 2131 patients [4.0%] in cohort 2) and was then assessed separately.

**Figure 1.**
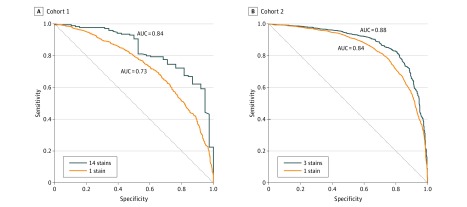
Prediction of Estrogen Receptor Positivity Using Deep Convolutional Neural Network The receiver operating characteristic curves for cohort 1 and cohort 2 were obtained by fitting the computed *r* score per patient to the estrogen receptor status (a single tissue microarray image or 3 tissue microarray images in cohort 2 and 14 images in cohort 1). The area under the receive operating characteristic (AUC) is indicated for each case.

The trained CNN was used to obtain *r* scores, per image and per patient, in 6-fold cross-validation (details in eMethods 5 in the Supplement). These scores were used to create receiver operating characteristics curves by fixing *T_l_* = *T_h_* and swiping their value between 0 and 1. For each value, the specificity and sensitivity were computed by comparing the resulting predictions to the ground-truth ER expressions (Figure 1). Overall, the deep CNN-based features had a better AUC for ER prediction than the feature extraction pipeline–based features. A combined score of multiple TMA images yielded better results than a single image. Given that cohort 2 included 10 times more patients than cohort 1, the better AUC for this cohort was not surprising.

We set the thresholds to *T_l_* = 0.25 and *T_h_* = 0.75, resulting in prediction of 105 of 207 validation patients (50.7%) in cohort 1 (positive predictive value, 97%; negative predictive value, 68%; accuracy, 91%) and 1059 of 2046 validation patients (51.8%) in cohort 2 (positive predictive value, 98%; negative predictive value, 76%; accuracy, 92%) and to *T_l_ = *0.50 and *T_h_ *= 0.50 (resulting in prediction of all patients) and summarized the results of CNN-based MBMP prediction of ER (eTable 3 in the Supplement). In addition, we summarized the concordance rates of MBMP (with thresholds *T_l_* = 0.25 and *T_h_* = 0.75) and IHC using different US Food and Drug Administration–approved antibody clones and the concordance rates of IHC and previously used ligand binding assays (Table). This analysis showed that with adequate sensitivity thresholds, MBMP had comparable accuracies to direct molecular assays for ER detection, with noninferiority to traditional IHC (positive predictive value, 91%-98%; negative predictive value, 51%-78%; accuracy, 81%-90%).

**Table.** Performance of MBMP and Comparison With Other Methods^a^

Source	Data Set	Assay Methods Compared (Antibody)	PPV, %	NPV, %	Sensitivity, %	Specificity, %	Accuracy, %
Proposed method	Cohort 1 (01-011)	MBMP and IHC (SP1)	98	68	93	90	92
Proposed method	Cohort 2 (02-008)	MBMP and IHC (SP1)	97	76	93	87	91
Cheang et al,^14^ 2006	Cohort 2 (02-008)	IHC (SP1) and DCC	98	62	86	92	87
Cheang et al,^14^ 2006	Cohort 2 (02-008)	IHC (1D5) and DCC	97	51	78	92	81
Cheang et al,^14^ 2006	Cohort 2 (02-008)	IHC (1D5) and IHC (SP1)	97	78	88	94	90
Barnes et al,^24^ 1996	Their own data set	LBA and IHC (1D5)	NA	NA	NA	NA	81
Regan et al,^25^ 2006	IBCSG	LBA and IHC (1D5)	NA	NA	NA	NA	88
Harvey et al,^26^ 1999	San Antonio tumor bank	LBA and IHC (1D5)	NA	NA	NA	NA	86
Hammond et al,^12^ 2010	IBCSG premenopausal	Primary institution by LBA/ELISA and central testing by IHC (1D5)	91	63	NA	NA	82
Hammond et al,^12^ 2010	IBCSG postmenopausal	Primary institution by LBA/ELISA and central testing by IHC (1D5)	93	73	NA	NA	88

Abbreviations: DCC, dextran-coated charcoal; ELISA, enzyme-linked immunosorbent assay; IBCSG, International Breast Cancer Study Group; IHC, immunohistochemistry; LBA, ligand binding assay; MBMP, morphological-based molecular profiling; NA, not applicable; NPV, negative predictive value; PPV, positive predictive value.

^a^ Concordance rates between MBMP low and high thresholds (low, 0.25; high, 0.75) and different criterion standard assays for estrogen receptor detection were obtained from Hammond et al^12^ and Chean et al.^14^ The statistical measures were computed considering the second method as the ground truth.

We used multiple logistic regression to assess the added value of the *r* scores in the context of other clinical and molecular factors (eTable 4 in the Supplement). In cohort 1, the obtained *r* scores were significantly associated with ER status (likelihood ratio χ^2^ = 28.81; *P* < .001) independent of prognosis and all other clinical and molecular features. In cohort 2, the *r* scores (likelihood ratio χ^2^ = 86.12; *P* < .001), PR (likelihood ratio χ^2^ = 251.03; *P* < .001), epidermal growth factor receptor (likelihood ratio χ^2^ = 33.48; *P* < .001), insulinlike growth factor type 1 receptor (likelihood ratio χ^2^ = 31.13; *P* < .001), GATA3 (likelihood ratio χ^2^ = 27.09; *P* < .001), αB-crystallin gene 4000 (likelihood ratio χ^2^ = 26.43; *P* < .001), P-cahedrin (likelihood ratio χ^2^ = 13.46; *P* = .001), p53 (likelihood ratio χ^2^ = 11.07; *P* = .003), and *HER4* (likelihood ratio χ^2^ = 10.51; *P* = .005) were each significantly associated with the ER status. The rest of the factors were not significant independent predictors of the ER status in this model.

### Performance and the Amount of Training and Validation Data

**Figure 2.**
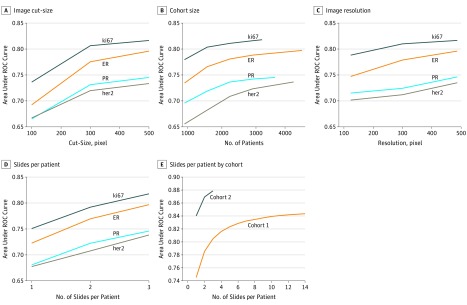
Amount of Data vs System Performance For cohort 2 (A-D), the resulting area under the receiver operating characteristics (ROC) curve (AUC) for prediction of Ki-67, estrogen receptor (ER), progesterone receptor (PR), and *ERBB2* status used the proposed logistic regression classifier. The AUC is plotted with respect to the biopsy cut size, the number of patients in the cohort, the image resolution, and the number of tissue microarray (TMA) slides per patient. For both cohorts (E), the resulting AUC for prediction of ER status used the proposed deep convolutional neural network. The AUC is plotted with respect to the number of TMA images per patient for cohorts 1 and 2. In cohort 2, 3 TMA images were available for each patient, whereas in cohort 1, 14 TMA images were available per patient.

The resulting AUC continuously improved without reaching saturation for each variable and biomarker, implying that training on more data would improve biomarker prediction accuracy (Figure 2). Unlike the other variables, the TMA-images-per-patient variable is changed at inference time. In agreement with Figure 1, increasing the number of images per patient markedly improved the system’s performance without the need to retrain the model for the logistic regression and for the CNN (Figure 2D and E). Unlike standard molecular assays, MBMP is a data-driven approach. This analysis showed the potential of MBMP to outperform traditional laboratory techniques for molecular quantitation, given enough data.

### MBMP's *r* Score and ER Expression in Breast Cancer

**Figure 3.**
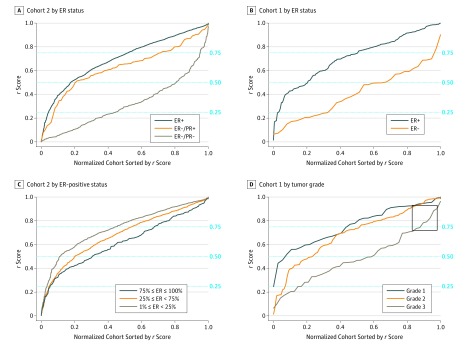
The Resulting *r* Scores for Prediction of Estrogen Receptor (ER) Positivity in All Patients The *r* scores were obtained using the proposed deep convolutional neural network. The horizontal axis represents the entire cohort population, normalized between 0 and 1, and sorted by the *r* score. The *r* scores are stratified by the ER status (A and B), by the percentage of cells expressing ER (only for patients with ER-positive tumor) (C), and by the tumor grade. Cases of high-grade malignant neoplasms for which the system could identify ER-associated morphological signal are boxed (D). PR indicates progesterone receptor.

The proposed CNN can be interpreted as a function that maps H&E-stained images to a score *r* in the interval (0,1), which measures the morphological signal indicative of molecular expression. Figure 3A and B demonstrate the positive association between the *r* scores and ER status. We applied the system to the excluded group of patients with ER-negative/PR-positive tumors in cohort 2 and added another curve for their resulting *r* scores (Figure 3A). Interestingly, the distribution of *r* scores for the ER-negative/PR-positive group resembled the distribution of ER-positive tumors (D_BC_ = 0.03) and not ER-negative/PR-negative tumors (D_BC_ = 0.25). In cohort 2, 1284 of 1558 patients with ER-positive tumors (82.4%) had *r* scores greater than 0.5, compared with 94 of 488 patients with ER-negative/PR-negative tumors (19.3%). 67 of 85 patients with ER-negative/PR-positive tumors (78.8%) had *r* scores greater than 0.5, almost similar to rates for patients with ER-positive tumors. This analysis supported the claim that among patients with ER-negative/PR-positive tumors, IHC failed to detect the ER.^2,23^

The *r* scores stratified by the percentage of cells expressing ER, for patients with ER-positive tumors, demonstrated a positive association with the percentage of ER-positive cells in the tissue (likelihood ratio χ^2^ = 53.64; *P* < .001) (Figure 3C). Thus, morphological surrogates for molecular expression could not only be identified but also could be quantified by MBMP, matching to ER’s occurrence in the tissue. This process might also explain why the patients with ER-negative/PR-positive tumors had lower *r* scores than patients with ER-positive tumors; failure to detect estrogen is more likely to occur when the percentage of ER-positive cells is low. The ER-positive cells had failed to be detected in these patients’ IHC-stained TMA images, and thus, their mean *r* scores were lower.

We next stratified the *r* scores of the patients in cohort 1 by their grade (Figure 3D). As expected, low-grade tumors had higher *r* scores than high-grade tumors. However, even in the rare cases of high-grade malignant neoplasms that are ER positive (box in Figure 3), the system identified morphological patterns that strongly imply an ER-positive status. This finding suggests that morphological patterns other than those reflected in the tumor grade are used by the system to determine ER expression.

### Estrogen Expression Could Be Learned From Stromal Regions

**Figure 4.**
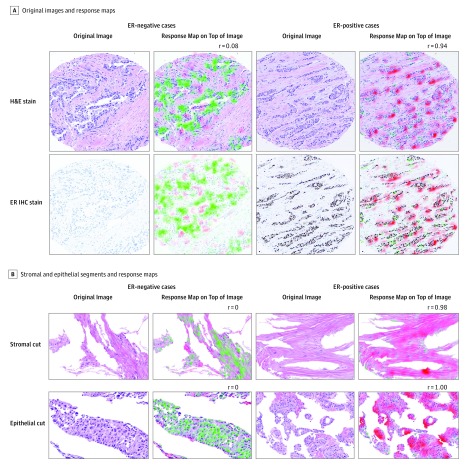
Hematoxylin-Eosin (H&E)–Stained Images With Corresponding Response Maps Patients with estrogen receptor (ER)–negative tumors are presented in the 2 left columns, and those with ER-positive tumors in the 2 right columns. Red regions correspond to morphological patterns that contribute to ER-positive prediction. Green regions correspond to morphological patterns that contribute to ER-negative prediction. Higher color intensity corresponds to a stronger contribution. The resulting *r* score is indicated for each case. The immunohistochemistry (IHC) images were never shown to the system.

Examination of the response maps did not reveal specific histological features that correlate to hormonal expression, such as inflammatory infiltrate or matrix variability. Unsurprisingly, prediction of ER status seemed to be learned based on the epithelial areas of the specimen (Figure 4). However, ER expression was also learned from stromal parts of the specimens. We used cutout stromal and epithelial regions of 243 test images from cohort 1 and applied the response map inference pipeline to the cutout segments independently. The prediction performance was obtained for the stromal regions (accuracy, 0.8; AUC, 0.75; balanced accuracy, 0.66) and for the epithelial regions (accuracy, 0.78; AUC, 0.77; and balanced accuracy, 0.69). We computed *P* values as the probability for a random classifier to obtain the indicated balanced accuracy or higher (stromal regions, *P* = .003; epithelial regions, *P* = .001). These correlations might help to explain previous findings suggesting that stromal morphology contains interpretable clues for patient prognosis.^27,28,29^

## Discussion

We have developed a computerized system for prediction of molecular markers of cancer by analysis of tissue histomorphology. For such a system to be feasible, a correlation must first be established between tissue morphology and molecular expression of the epitope in question. Our analysis of breast cancer tissue specimens revealed that all the assayed biomarkers had identifiable signatures in tissue morphology, regardless of the marker’s subcellular (nuclear, cytoplasmic, or membranal) or tissue (stromal or epithelial compartments) localization (eTable 2 in the Supplement). Moreover, biomarkers that were more likely to be influential in the biology of breast cancer had the highest prediction accuracies. This finding demonstrated the credibility of the results, because the molecular pathways that govern the tumor’s behavior were expected to leave a more profound histological fingerprint.

We then tailored deep CNN to predict biomarker expression from H&E-stained histological images and used ER as a showcase on which to test the system. Our results show that for at least half of the patients, MBMP had comparable accuracy to IHC in predicting ER expression (Table). Moreover, the *r* scores were correlated with the percentage of ER-presenting cells as determined by IHC, demonstrating that the morphological signal indicative of molecular expression could be not only identified but quantified. The ability to identify patients who may benefit from antihormonal therapy by IHC had a marked effect on the survival of patients with breast cancer.^30^ However, IHC has inherent and technical limitations that may come down to considerable inconsistencies in ER evaluation.^12,13,14,15,31^ In contrast, MBMP escapes technical issues such as fixation or antigen retrieval, obsoletes the need for subjective human interpretation, and avoids false-negative findings due to splice variants missing the antibody binding site. Such advantages of MBMP over IHC could be demonstrated for the group with ER-negative/PR-positive tumors, who are widely considered to have an ER-positive phenotype but with false-negative findings of IHC staining.^2,23^ Our results indicated that patients with ER-negative/PR-positive tumors share more similarities with patients with ER-positive tumors than with their ER-negative/PR-negative counterparts, in support of antihormonal therapy for this group of patients.

The interpretability problem of artificial neural networks poses major challenges and complicates supervision of the system aimed to identify prediction errors.^32,33^ To trace the learning, we used an approach that highlights hot spots in the image, from which MBMP learned the most to reach its conclusion. The response maps we created from segmented images demonstrate that analysis of the tumor stroma independently contributed to the prediction of ER receptor expression. These results may explain findings by Beck et al^11^ that prognosis can be predicted by analysis of stromal elements, because patients with ER-positive tumors generally have better prognosis. Although we could not identify meaningful histomorphological structures that the system used to make its prediction, the response maps may provide a future avenue to supervise the credibility of the system’s responses through dedicated analysis of the predictive area in each image.

### Limitations

The data set used for this work was unique in its quality and quantity, allowing successful implementation of a data-thirsty method such as CNN. However, the data set itself was the major caveat of this work. It originated from a single institution in Canada, included only TMA images rather than whole-slide specimens, and may have been too small to fully exploit the potential of neural networks. Thus, for MBMP to be universally applicable, a multi-institutional shared database of annotated H&E-stained images needs to be erected, with suitable mechanisms for data anonymization and sharing.^34,35^ For newly added cohorts, a system calibration phase will be needed, which consists of training another cohort-specific ResNet on a set of institution-scanned H&E-stained images and their corresponding annotations. The TMAs may be simpler to analyze than whole-slide images because humans predefined regions of interest to be studied. However, because more sample images and a larger cut size were associated with superior performance, and because the system learned from the stromal regions and not only from cancerous structures, it is safe to assume that the use of whole-slide images would improve the performance of the system. Moreover, current machine learning tools can now automatically identify cancerous regions in whole-slide images noninferiorly to pathologists.^36,37^ The sheer amount of data used for neural network learning is probably the most influential factor for successful biomarker predictions.

## Conclusions

As our understanding of molecular origin of diseases expands, an increasing number of molecular markers are expected to be quantified in each pathologic specimen handled by laboratories. We envision MBMP technology playing a pivotal role in the pathologic processing and analysis workflow. As in the case of ER, other molecular markers could be accurately predicted in parallel. For those who obtain high confident *r* scores, molecular identification using direct assays might be unnecessary, because MBMP has noninferior accuracy to IHC in this population. Morphological-based molecular profiling could also be used as a screening phase that predicts activation of culprit molecular pathways in cancer, assisting pathologists in the choice of downstream molecular analysis. Finally, in the developing world and in circumstances in which reliable IHC is out of reach, MBMP could serve as an essential tool for physicians to guide the choice of therapeutic regimens and choose targeted drugs.

## Notes

Supplement. **eMethods 1.** Additional Information Regarding the Digital Scanner, Eligible Patients, and the Database Source **eMethods 2.** Practical Considerations for Combining Image Scores Into a Single Patient Score **eMethods 3.** Amount of Data vs the System’s Performance: Additional Details for Reproducibility **eMethods 4.** A Full Description of the MBMP Process **eMethods 5.** Additional Details and Considerations Regarding the Train and Test Partitions **eFigure 1.** Logistic Regression Training Model **eFigure 2.** Feature Extraction Pipeline **eFigure 3.** Deep Convolutional Network Model for Training and Inference **eFigure 4.** Response Map Inference Pipeline **eTable 1.** Cut Points, Number of Patients, and Number of H&E Images **eTable 2.** Association of Biomarker Expression and Tumor Morphology **eTable 3.** Performance of Morphological-Based Molecular Profiling **eTable 4.** Multiple Logistic Regression
