# Error in Figure 3C

**DOI:** 10.1001/jamanetworkopen.2019.11247

**Published:** 2019-08-16

In the Original Investigation titled “Artificial Intelligence Algorithms to Assess Hormonal Status From Tissue Microarrays in Patients With Breast Cancer,”^1^ published July 26, 2019, the labeling of the tan and navy lines in the key of Figure 3C were inadvertently switched. The navy line should indicate “1% ≤ ER < 25%,” and the tan line should indicate “75% ≤ ER ≤ 100%.” This article has been corrected.^1^
