# Supplementary material for: Artificial Intelligence Algorithms to Assess Hormonal Status From Tissue Microarrays in Patients With Breast Cancer
Source: JAMA Netw Open. 2019 Jul 26;2(7):e197700. doi: 10.1001/jamanetworkopen.2019.7700 (PMC6661721; doi:10.1001/jamanetworkopen.2019.7700)
Supplement: Supplement. — eMethods 1. Additional Information Regarding the Digital Scanner, Eligible Patients, and the Database Source eMethods 2. Practical Considerations for Combining Image Scores Into a Single Patient Score eMethods 3. Amount of Data vs the System’s Performance: Additional Details for Reproducibility eMethods 4. A Full Description of the MBMP Process eMethods 5. Additional Details and Considerations Regarding the Train and Test Partitions eFigure 1. Logistic Regression Training Model eFigure 2. Feature Extraction Pipeline eFigure 3. Deep Convolutional Network Model for Training and Inference eFigure 4. Response Map Inference Pipeline eTable 1. Cut Points, Number of Patients, and Number of H&E Images eTable 2. Association of Biomarker Expression and Tumor Morphology eTable 3. Performance of Morphological-Based Molecular Profiling eTable 4. Multiple Logistic Regression [file jamanetwopen-2-e197700-s001.pdf]

# Supplementary Online Content

Shamai G, Binenbaum Y, Slossberg R, Duek I, Gil Z, Kimmel R. Artificial intelligence algorithms to assess hormonal status from tissue microarrays in patients with breast cancer. *JAMA Netw Open.* 2019;2(7):e197700. doi:10.1001/jamanetworkopen.2019.7700

**eMethods 1.** Additional Information Regarding the Digital Scanner, Eligible Patients, and the Database Source

**eMethods 2.** Practical Considerations for Combining Image Scores Into a Single Patient Score

**eMethods 3.** Amount of Data vs the System's Performance: Additional Details for Reproducibility

**eMethods 4.** A Full Description of the MBMP Process

**eMethods 5.** Additional Details and Considerations Regarding the Train and Test Partitions

**eFigure 1.** Logistic Regression Training Model

**eFigure 2.** Feature Extraction Pipeline

**eFigure 3**. Deep Convolutional Network Model for Training and Inference

**eFigure 4**. Response Map Inference Pipeline

**eTable 1.** Cut Points, Number of Patients, and Number of H&E Images

**eTable 2.** Association of Biomarker Expression and Tumor Morphology

**eTable 3.** Performance of Morphological-Based Molecular Profiling

**eTable 4**. Multiple Logistic Regression

This supplementary material has been provided by the authors to give readers additional information about their work.

© 2019 Shamai G et al. *JAMA Network Open.*

**eMethods 1.** Additional Information Regarding the Digital Scanner, Eligible Patients, and the Database Source

The patients in Cohort 1 were obtained from archival cases at Vancouver General Hospital between 1974 and 1995. The tumor specimens of the patients in Cohort 2 were processed by a central laboratory at Vancouver General Hospital between 1986 and 1992. In Cohort 1, any patient that was annotated for ER status was eligible for the study and analysis. In Cohort 2, any patient that had unequivocal annotation of a certain biomarker was eligible for the study and analysis of this biomarker (4,745 of the 4,944 patients had annotation to at least one biomarker). The median age at diagnosis was 61 years for Cohort 1 (412 patients) and 62 years for Cohort 2 (4,944 patients), and the median follow-ups were 12.0 and 12.4 years, respectively. The number of eligible patients, the annotated biomarkers, and the binary cut-point used in this paper are summarized in eTable 1. The group of ER-/PR+ patients in Cohort 2 was analyzed independently when applying the system. The TMA slides contain 0.6-mm-diameter cores and were scanned using the Bacus Laboratories, Inc. Slide Scanner (Bliss) scanner at a resolution of $2256 \times 1440$ pixels. In our data processing, a square section of $1440 \times 1440$ was cut from the middle of each images, and then all images were resized to a resolution of $512 \times 512$. No additional image processing was done prior to the CNN model and feature extraction pipeline. All data and annotations can be found on http://bliss.gpec.ubc.ca/ and http://www.gpecimage.ubc.ca/.

**eMethods 2.** Practical Considerations for Combining Image Scores Into a Single Patient Score

The logistic regression obtains a set of features encoding a single H&E image and outputs its *r* score. Given *k* images with output scores $r_1, r_2, \ldots, r_k$ belonging to the same patient, we computed the combined patient's *r* score similarly to [1]. Namely, we averaged the feature set across all images belonging to this patient and then applied the logistic regression. This could be interpreted as concatenating all TMA images of the patient and applying the pipeline on the concatenated image. In practice, this procedure is equivalent to averaging the prediction scores $l_i$ before the sigmoid operation $r_i = \frac{1}{1+e^{-l_i}}$ in the logistic regression, which is equivalent to computing the *r* score of a patient as

$$r = \frac{1}{1+e^{\frac{1}{k}\sum_{i=1}^{k}\ln\left(\frac{1}{r_i - 1}\right)}}.$$

**eMethods 3.** Amount of Data vs the System's Performance: Additional Details for Reproducibility

To change the resolution according to the parameter *R*, all images were resized to $R \times R$ pixels using a standard bicubic interpolation. To change the cut size according to the parameter *C*, a square of size $C \times C$ pixels was cut out from the center of each image. To change the number of TMA images per patient according to the parameter *S*, for each patient, *S* of the images belonging to the patient were selected randomly. Then, only these images were taken into consideration in the prediction process. To change the number of patients according to the parameter *P*, *P* patients were selected randomly to form a new dataset. Each of the parameters *P,R,C,S* was changed at a time while the rest were kept fixed at their maximal values. Since for the parameters *P* and *S* a random selection was used in the process, we repeated the experiment *N* times and averaged the results. The number of repetitions *N* was set such that the 95% confidence interval of the resulting average was less than 1% around the average value.

**eMethods 4.** A Full Description of the MBMP Process

The MBMP process consists of four stages. The first three stages are aimed at constructing the model, and are done only once per cohort, per cancer type, and per marker. The fourth stage is a decision module that outputs the final prediction based on the patient's H&E-stained images.

1. Data collection

In the first stage, digital H&E-stained images should be collected of a set of patients from the cohort. Our experiments show that with better resolution, larger cut-size, larger number of patients and especially larger number of TMA images per patient, the system's performance is likely to improve. In our work we used TMA images with

© 2019 Shamai G et al. *JAMA Network Open.*

size 512 × 512. If the work is done on whole slide images (WSI) and not on TMAs, we recommend extracting non-overlapping patches from each WSI and treat them as multiple TMA images.
The molecular expression of the molecule in question should be collected for each patient. In our work we focus on binary labels (positive/negative status). However, we showed that the actual percentage is expressed as well and not only the binary status, and thus could probably be predicted given enough data. The molecular expression labels of the patients are then assigned to their H&E images.
The patients (and their images) in the collected dataset should be split to a training and validation set. For both cohorts in our work, we used 5/6 of the patients in the training set and the rest of the 1/6 patients for the validation set. A different setting may be more beneficial for different types datasets.

2. Training or using an existing cohort-specific and a cross-cohort ResNets
Once H&E images and their corresponding molecular expression labels are collected, the next step is to train the cohort-specific ResNet to predict the labels from the H&E images. The architecture of the ResNet we used is described in eFigure 3A. We used the common ResNet architecture [2] without any architectural changes. The ResNet should be trained using the training set.
If a Cross-Cohort ResNet that was already trained on several other cohorts is available, it might be sufficient to use the existing one. Otherwise, to improve the performance, another Cross-Cohort ResNet should be trained to predict the label from H&E belonging to all available cohorts (including the current one). This ResNet should not be trained on the validation set of the current cohort.

3. Training the logistic regression on a validation set
In the next step, the two ResNets should be applied to the images belonging to the validation set of patients that were not seen so far. The 64 features of each ResNet should be concatenated into a vector of 128 features per image, according to the inference model in eFigure 3B. A logistic regression should then be trained on these features to extract the final label.

4. Inference and decision
The previous stages should be done only once per cohort, cancer type, and molecule in question. Given H&E images of a test patient that was never seen by the system, the two trained ResNets should be applied to all images to extract 128 features from each. These features should be averaged to obtain one vector of 128 features. Then, the trained logistic regression should be applied to these features (eFigure 3B). The output of the logistic regression is a score $r$ that denotes the probability of the molecule in question is expressed for this patient.
The molecular expression should be predicted as negative for $r < T_l$, positive for $T_h < r$, and inconclusive for $T_l < r < T_h$. These thresholds should hold the condition $0 < T_l \leq T_h < 1$, and should be determined according to the desired accuracy and screening process (see Methods and Results in the main text).

**eMethods 5.** Additional Details and Considerations Regarding the Train and Test Partitions

For the logistic regression, the performance was assessed by 10-fold cross-validation model, where in each fold the system was trained on 9 subsets of the patients and tested on the held-out patients. This partition setting exploited 9/10=90% of the data for training the model, while statistical outcomes were obtained and evaluated for all cases. The deep CNN was trained and asses by 6-fold cross validation, where in each fold, 5 groups were used to train the ResNet units. The trained ResNet units were applied to the remainder group to extract the features. Then, these features were used to train and test the logistic regression unit in an inner 10-fold cross-validation manner. This partition setting exploited 5/6=83.3% of the data for training the CNN model and 9/10*1/6 = 15% of the data for training the logistic regression on the CNN-based features. We randomly chose 3 out of the 6 folds and performed the training and inference process. Thus, statistical outcomes were obtained and evaluated for 3/6=50% of the cases. The performance of each of the presented systems was assessed on images of previously unseen patients. Extra care was taken to ensure that any data belonging to the test patients, including their H&E-stained TMA images, IHC-stained TMA images, and biomarker annotations, were concealed from the systems during their training.

© 2019 Shamai G et al. *JAMA Network Open.*

## **eFigure 1.** Logistic Regression Training Model

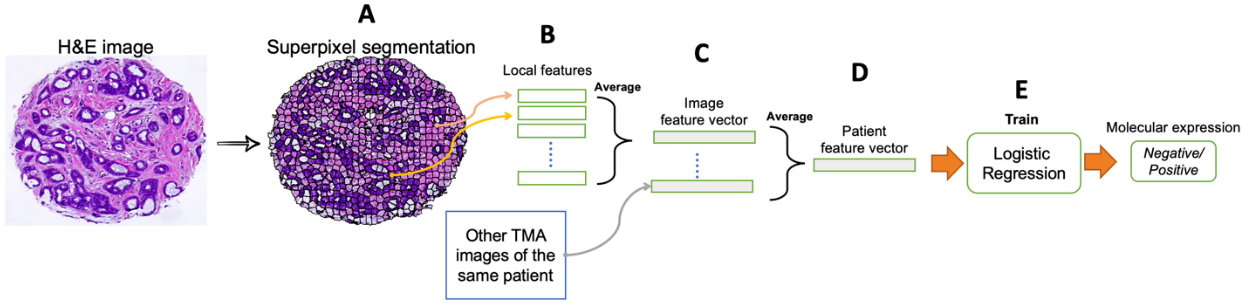


(**A)** The H&E image is segmented into non-overlapping superpixel patches, and (**B**) local features are extracted from each patch using different arithmetic operations (eFigure 2). (**C**) The features are averaged across all patches, as well as (**D**) across all other TMA images that belong to the same patient, to obtain a final patient feature vector. (**E**) An $L_1$ regularized logistic regression is trained to predict biomarker expression from its feature vector.

© 2019 Shamai G et al. *JAMA Network Open.*

## **eFigure 2.** Feature Extraction Pipeline

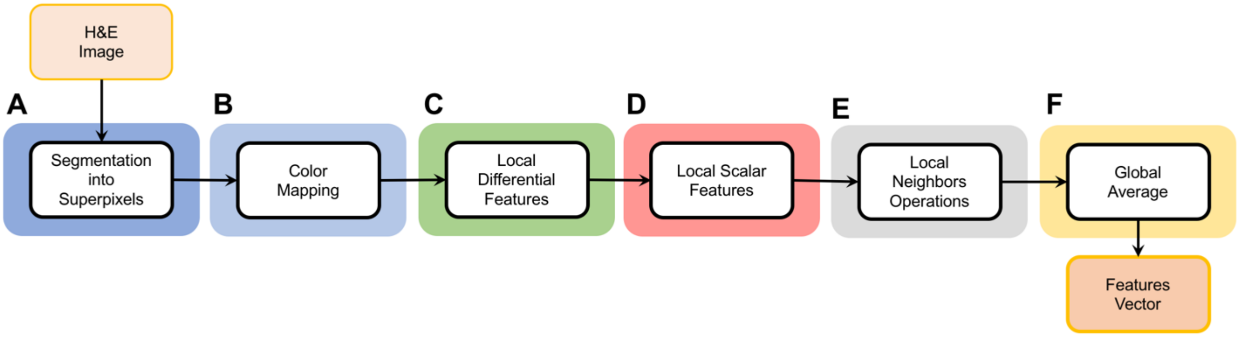


(**A**) The H&E image is segmented into non-overlapping small patches termed superpixels **[3]**. (**B**) The H&E image is decoupled into red, green, and blue (RGB) channels such that each channel was a two-dimensional array. In the same manner, it is decoupled into hue, saturation, and value channels. Lastly, it is decoupled into hematoxylin and eosin channels, according to the color unmixing model previously described **[4]**. Overall, the H&E image is transformed into 8 two-dimensional images. (**C**) Gradient (magnitude) and Laplacian of Gaussians (LoG) are widely used operators that involve convolving the image with appropriate filters to produce another image that typically can be used for finding edges and extracting interesting structures like blobs. Here, each one of these two operators are applied to each one of the 8 channels to obtain an overall of 8*3=24 two-dimensional channel images (including the original 8 channels). Note that these channels inherit the superpixel segmentation of stage (**A**). (**D**) Unlike operators which map an image to an image, the local operators in this stage map each superpixel patch into a scalar value. We used 9 such operators - min, max, mean, median, standard deviation (STD), the standard first order histogram features skewness, energy, and entropy, and distance to nearest patch neighbor (between centroids). In this manner, 24*9 = 216 scalar values are extracted from each patch. (**E**) The features of each patch are integrated with the features of its 8 nearest patch neighbors (measured via distance between their centroids), by applying 6 operators - min, max, mean, median, sum, and STD, overall obtaining 216*6=1,296 scalar values per patch. (**F**) Finally, each feature is averaged across all patches to obtain a final vector of 1,296 scalar values that encodes the entire image.

© 2019 Shamai G et al. *JAMA Network Open.*

**eFigure 3**. Deep Convolutional Network Model for Training and Inference

**A**. ResNet architecture

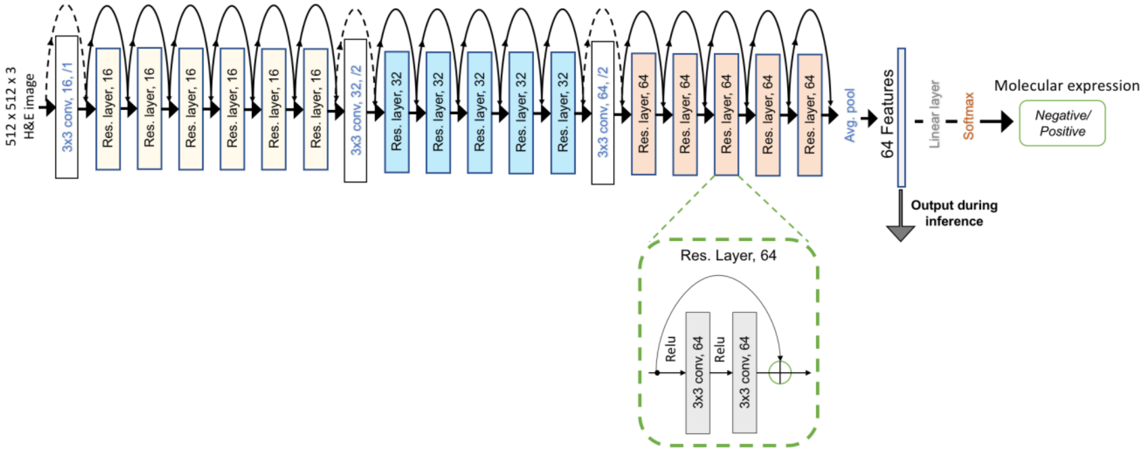


**B**. Inference pipeline

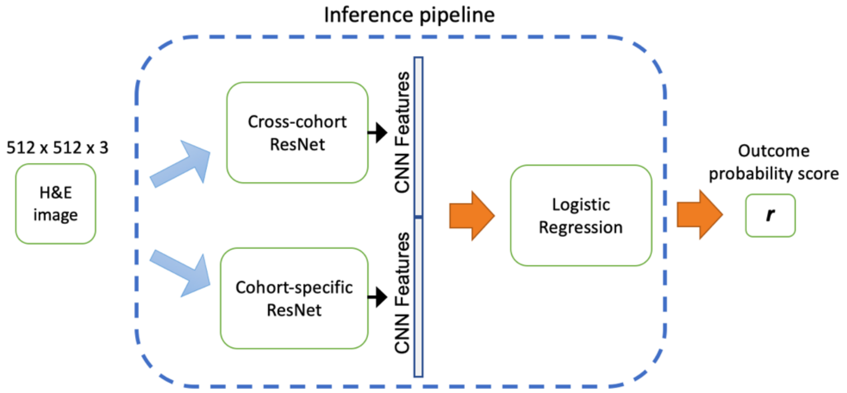


**(A)** Deep convolutional residual network (ResNet) architecture. The architecture of the residual layer is shown inside the dashed green frame. During inference, we use the common practice of omitting the final linear layer to obtain the 64-feature vector produced by the network. **(B)** The CNN inference model. To compensate for cohort diversity, the inference pipeline (blue frame) includes two ResNet units, termed as the cross-cohort ResNet and the cohort-specific ResNet, that were each trained to predict the molecular expression. The cross-cohort Resnet was trained on H&E images from both Cohort 1 and Cohort 2 and a specific-cohort ResNet was trained for each cohort independently, i.e., only on H&E images belonging to patients from this cohort. The features of both ResNets are aggregated and used as an input to an $L_1$ regularized logistic. The final output score *r*, which is a derivative of the commonly used logistic regression model, indicates the predicted probability for positive molecular expression.

© 2019 Shamai G et al. *JAMA Network Open.*

**eFigure 4**. Response Map Inference Pipeline

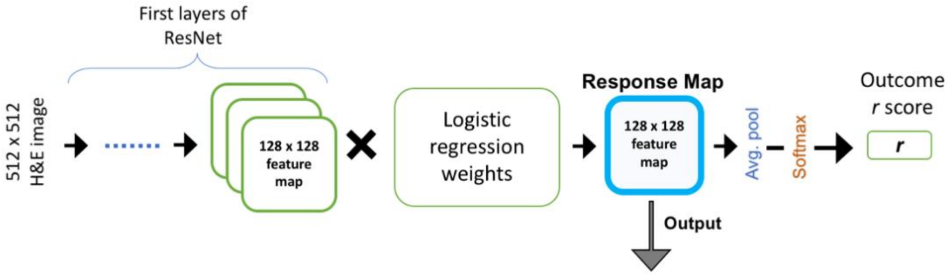


A test H&E is passed through the first layers of the trained network to obtain 64 128 × 128 feature maps. Previously (eFigure 3), the feature maps were passed through an average pooling, linear layer and then softmax to obtain the final *r* score. Here, instead, these maps are weighted-averaged with the weights of the trained logistic regression to obtain a single map, termed as the *response map*. Since weighted average and average pooling are linear operations, changing their order should not influence the final *r* score. i.e, performing average pooling and softmax on the response map results in the same *r* score obtained in the previous pipeline. In the resulting response map, areas with positive values in the correspond to morphology patterns that contribute to positive prediction, and vice versa. Since this pipeline is fully based on spatial-invariant operations such as convolutions, the output response map locally matched the H&E image.

© 2019 Shamai G et al. *JAMA Network Open.*

**eTable 1.** Cut Points, Number of Patients, and Number of H&E Images

| Biomarker | Negative | Positive | #Patients | #H&E images |
|---|---|---|---|---|
| **A. Cohort 2** | | | | |
| Ki-67 | Score < 10% | Score >= 14% | 3,292 | 9,876 |
| EGFR | Score = 0% | Score >= 1% | 3,905 | 11,715 |
| ER | Score = 0% | Score >= 1% | 4,521 | 13,563 |
| FOXP3-STIL | STIL < 3 | STIL >= 3 | 3,277 | 9,831 |
| CD8-STIL | STIL < 3 | STIL >= 3 | 3,905 | 11,715 |
| CA9 | Score = 0% | Score >= 1% | 4,150 | 12,450 |
| IGF-1R | Score < 67% | Score >= 67% | 1,948 | 5,844 |
| FOXP3-ITIL | ITIL < 2 | ITIL >= 2 | 3,277 | 9,831 |
| PR | Score = 0% | Score >= 1% | 3,691 | 11,073 |
| HER2 | Score < 10%, OR<br>10% <= Score < 30%<br>and<br>fish amplification < 1.8 | Score >= 30%, OR<br>10% <= Score < 30%<br>and<br>fish amplification > 2.2 | 4,264 | 12,789 |
| P53 | Score < 10% | Score >= 10% | 4,462 | 13,386 |
| CD8-ITIL | STIL < 1 | ITIL >= 1 | 3,905 | 11,715 |
| CK56 | Score < 20% | Score >= 20% and strong staining | 3,828 | 11,484 |
| RET | Score = 0% | Score >= 1% | 3,634 | 10.902 |
| HER3 | Score < 20% | Score >= 20% | 3,516 | 10,584 |
| P-CAHEDRIN | Score < 30% | Score >= 70% | 4,273 | 12,819 |
| MDM2 | Score = 0% | Score >= 1% | 1,979 | 5,937 |
| CRYAB4000 | Score < 30% | Score >= 30% | 3,704 | 11,112 |
| GATA3 | Score < 5% | Score >= 5% | 3,539 | 10,617 |
| HER4 | Score = 0% | Score >= 1% | 3,824 | 11,472 |
| C-KIT | Score = 0% | Score >= 3% | 4,035 | 12,105 |
| **B. Cohort 1** | | | | |
| ER | Score < 10% | Score >= 10% | 412 | 5,768 |

The molecular biomarkers in Cohort 2 (**A**) and Cohort 1 (**B**) are shown with their corresponding cut-off points. The number of patients participating in the experiment varies for different biomarkers and, together with the total number of H&E images, is indicated for each biomarker.The cut-off points were obtained from the papers describing expression of these biomarkers for Cohort 1 and Cohort 2 http://www.gpecimage.ubc.ca/. (ITILs - intratumoral tumor-infiltrating lymphocytes. STILs - stromal tumor-infiltrating lymphocytes).

© 2019 Shamai G et al. *JAMA Network Open.*

**eTable 2.** Association of Biomarker Expression and Tumor Morphology

| **Number** | **Biomarker** | **AUC** | **BACC** | ***P* value** |
|---|---|---|---|---|
| **1** | Ki-67 | 0.82 | 0.74 | <0.001 |
| **2** | EGFR | 0.82 | 0.74 | <0.001 |
| **3** | ER | 0.8 | 0.73 | <0.001 |
| **4** | FOXP3-STIL | 0.78 | 0.7 | <0.001 |
| **5** | CD8-STIL | 0.77 | 0.69 | <0.001 |
| **6** | CA9 | 0.76 | 0.7 | <0.001 |
| **7** | IGF-1R | 0.76 | 0.69 | <0.001 |
| **8** | FOXP3-ITIL | 0.76 | 0.7 | <0.001 |
| **9** | PR | 0.75 | 0.69 | <0.001 |
| **10** | HER2 | 0.74 | 0.68 | <0.001 |
| **11** | P53 | 0.73 | 0.68 | <0.001 |
| **12** | CD8-ITIL | 0.72 | 0.66 | <0.001 |
| **13** | CK56 | 0.71 | 0.67 | <0.001 |
| **14** | RET | 0.7 | 0.65 | <0.001 |
| **15** | HER3 | 0.7 | 0.65 | <0.001 |
| **16** | P-CAHEDRIN | 0.69 | 0.64 | <0.001 |
| **17** | MDM2 | 0.68 | 0.64 | <0.001 |
| **18** | CRYAB4000 | 0.68 | 0.62 | <0.001 |
| **19** | GATA3 | 0.68 | 0.62 | <0.001 |
| **20** | HER4 | 0.67 | 0.63 | <0.001 |
| **21** | C-KIT | 0.66 | 0.61 | <0.001 |

The resulting AUC, BACC and *P* values of the $L_1$ regularized regression classification are shown for each one of the 19 tested molecular biomarkers. The lymphocyte markers CD8 and FOXP3 were further stratified to intratumoral tumor-infiltrating lymphocytes (ITILs) and stromal tumor-infiltrating lymphocytes (STILs).

© 2019 Shamai G et al. *JAMA Network Open.*

**eTable 3.** Performance of Morphological-Based Molecular Profiling
Statistical summary of the prediction performance of CNN based MBMP with different thresholds for both cohorts, compared to IHC done with SP1 antibody.

| | | IHC (SP1) | | | | |
|---|---|---|---|---|---|---|
| **Method and cohort** | **Measure** | **Negative** | **Positive** | **Total** | **Predictive Value** | **Performance** |
| **MBMP (*$T_l$ = 0.5, $T_h$ = 0.5*)** | | | | | | |
| **Cohort 2** | | | | | | |
| | Negative | 394 | 274 | 668 | 59% | |
| | Positive | 94 | 1284 | 1378 | 93% | |
| | Total classified | 488 | 1558 | 2046 (100%) | | |
| | BACC | | | | | 82% |
| | ACC | | | | | 82% |
| **Cohort 1** | | | | | | |
| | Negative | 25 | 35 | 60 | 42% | |
| | Positive | 13 | 134 | 147 | 91% | |
| | Total classified | 38 | 169 | 207 (100%) | | |
| | BACC | | | | | 73% |
| | ACC | | | | | 77% |
| **MBMP (*$T_l$ = 0.25, $T_h$ = 0.75*)** | | | | | | |
| **Cohort 2** | | | | | | |
| | Negative | 217 | 69 | 286 | 76% | |
| | Positive | 24 | 749 | 773 | 97% | |
| | Total classified | 241 | 818 | 1059 (52%) | | |
| | BACC | | | | | 91% |
| | ACC | | | | | 91% |
| **Cohort 1** | | | | | | |
| | Negative | 13 | 6 | 19 | 68% | |
| | Positive | 2 | 84 | 86 | 98% | |
| | Total classified | 15 | 90 | 105 (51%) | | |
| | BACC | | | | | 90% |
| | ACC | | | | | 92% |

© 2019 Shamai G et al. *JAMA Network Open.*

**eTable 4**. Multiple Logistic Regression
A multiple logistic regression model was fitted with the likelihood-ratio test to the ER status, using all other clinical and molecular measurements of Cohort 2 (**A**) and Cohort 1 (**B**) as independent variables. (ITILs - intratumoral tumor-infiltrating lymphocytes; STILs - stromal tumor-infiltrating lymphocytes).

| **Variable** | **L-R Chi-square** | ***P* value** |
|---|---|---|
| **A. Cohort 2** | | |
| PR | 251.03 | <0.001 |
| MBMP | 86.12 | <0.001 |
| EGFR | 33.48 | <0.001 |
| IGF-1R | 31.13 | <0.001 |
| GATA3 | 27.09 | <0.001 |
| CRYAB4000 | 26.43 | <0.001 |
| P-CAHEDRIN | 13.46 | 0.001 |
| P53 | 11.07 | 0.003 |
| HER4 | 10.51 | 0.005 |
| RET | 8.21 | 0.02 |
| HER2 | 8.08 | 0.02 |
| CK56 | 6.42 | 0.04 |
| Ki-67 | 5.21 | 0.07 |
| CD8-ITIL | 4.18 | 0.12 |
| CA9 | 3.61 | 0.16 |
| FOXP3-ITIL | 1.56 | 0.45 |
| HER3 | 1.44 | 0.48 |
| FOXP3-STIL | 0.33 | 0.56 |
| C-KIT | 1.01 | 0.60 |
| CD8-STIL | 0.13 | 0.70 |
| MDM2 | 0.36 | 0.83 |
| **B. Cohort 1** | | |
| MBMP | 26.81 | <0.001 |
| Time to death | 3.57 | 0.05 |
| Grade | 5.48 | 0.06 |
| Age | 1.61 | 0.20 |
| Tumor Size | 0.90 | 0.34 |
| Lymph Node Status | 0.85 | 0.35 |
| Mastectomy | 0.16 | 0.68 |

© 2019 Shamai G et al. *JAMA Network Open.*

## References

[1] A. H. Beck, A. R. Sangoi, S. Leung, R. J. Marinelli, T. O. Nielsen, M. J. Van De Vijver, R. B. West, M. Van De Rijn and D. Koller, "Systematic analysis of breast cancer morphology uncovers stromal features associated with survival," *Science translational medicine*, vol. 3, pp. 108ra113--108ra113, 2011.

[2] K. He, X. Zhang, S. Ren and J. Sun, "Deep residual learning for image recognition," in *Proceedings of the IEEE conference on computer vision and pattern recognition*, 2016.

[3] R. Achanta, A. Shaji, K. Smith, A. Lucchi, P. Fua, S. Süsstrunk and others, "SLIC superpixels compared to state-of-the-art superpixel methods," *IEEE transactions on pattern analysis and machine intelligence*, vol. 34, pp. 2274-2282, 2012.

[4] A. C. Ruifrok, D. A. Johnston and others, "Quantification of histochemical staining by color deconvolution," *Analytical and quantitative cytology and histology*, vol. 23, pp. 291-299, 2001.

© 2019 Shamai G et al. *JAMA Network Open.*
